# Axonal loss in the multiple sclerosis spinal cord revisited

**DOI:** 10.1111/bpa.12516

**Published:** 2017-05-07

**Authors:** Natalia Petrova, Daniele Carassiti, Daniel R. Altmann, David Baker, Klaus Schmierer

**Affiliations:** ^1^ Blizard Institute (Neuroscience), Barts and the London School of Medicine & Dentistry Queen Mary University of London London UK; ^2^ London School of Hygiene & Tropical Medicine London UK; ^3^ Neurosciences Clinical Academic Group the Royal London Hospital, Barts Health NHS Trust London UK

**Keywords:** atrophy, axons, demyelination, multiple sclerosis, spinal cord

## Abstract

Preventing chronic disease deterioration is an unmet need in people with multiple sclerosis, where axonal loss is considered a key substrate of disability. Clinically, chronic multiple sclerosis often presents as progressive myelopathy. Spinal cord cross‐sectional area (CSA) assessed using MRI predicts increasing disability and has, by inference, been proposed as an indirect index of axonal degeneration. However, the association between CSA and axonal loss, and their correlation with demyelination, have never been systematically investigated using human *post mortem* tissue. We extensively sampled spinal cords of seven women and six men with multiple sclerosis (mean disease duration= 29 years) and five healthy controls to quantify axonal density and its association with demyelination and CSA. 396 tissue blocks were embedded in paraffin and immuno‐stained for myelin basic protein and phosphorylated neurofilaments. Measurements included total CSA, areas of (i) lateral cortico‐spinal tracts, (ii) gray matter, (iii) white matter, (iv) demyelination, and the number of axons within the lateral cortico‐spinal tracts. Linear mixed models were used to analyze relationships. In multiple sclerosis CSA reduction at cervical, thoracic and lumbar levels ranged between 19 and 24% with white (19–24%) and gray (17–21%) matter atrophy contributing equally across levels. Axonal density in multiple sclerosis was lower by 57–62% across all levels and affected all fibers regardless of diameter. Demyelination affected 24–48% of the gray matter, most extensively at the thoracic level, and 11–13% of the white matter, with no significant differences across levels. Disease duration was associated with reduced axonal density, however not with any area index. Significant association was detected between focal demyelination and decreased axonal density. In conclusion, over nearly 30 years multiple sclerosis reduces axonal density by 60% throughout the spinal cord. Spinal cord cross sectional area, reduced by about 20%, appears to be a poor predictor of axonal density.

**Abbreviations**
aCST: cortico‐spinal tract area
CDD: chronic disease deterioration
CoV: coefficient of variation
CSA: cross sectional area
CST: Lateral cortico‐spinal tract
EDSS: expanded disability status scale
GM: gray matter
MBP: myelin basic protein
WM: white matter.

## INTRODUCTION

Multiple sclerosis (MS) is a common inflammatory demyelinating disease of the central nervous system leading to chronic disability 40. The most evident pathologic findings in the CNS of people with multiple sclerosis (pwMS) include focal white matter (WM) demyelination and remyelination, inflammation, a variable degree of axonal preservation and loss, and gliosis 35 as well as extensive gray matter (GM) tissue damage 34.

The spinal cord is the most frequent location of clinically‐apparent pathology at first presentation in pwMS 42. It is also a common manifestation site of chronic disease deterioration (CDD) leading to loss of urinary and fecal sphincter control as well as progressive motor and sensory dysfunction of the limbs and trunk resembling the clinical syndrome of progressive myelopathy 41.

Although inflammation and demyelination are important features throughout the disease course, degeneration of chronically demyelinated axons is considered the major pathological correlate of CDD 6. CDD may, at least in part, be mediated by mechanisms independent of the inflammatory penumbra associated with relapsing MS 1, 3, 18, 54. The reduction of spinal cord cross‐sectional area (CSA), measured using magnetic resonance imaging (MRI), is widely considered an indirect measure of axonal degeneration 31, 36, 56.

Though axonal loss has been described in MS for over 160 years 8, 38, a number of fundamental issues regarding axonal pathology in the chronic MS spinal cord have remained unclear. First, the reported degree of axonal loss after a life with MS has been highly variable ranging from 19 to 60% 12, 19, 37. Second, axonal loss in the MS cortico‐spinal tracts (CSTs) was found to be size‐selective, with small diameter fibers more affected than large ones 12. However, examination of the proportional rather than the absolute loss has, to our knowledge, never been undertaken 12, 19, 51. Thirdly, the distribution of lesions, degree of axonal loss, reduction of CST area (aCST) 12 and spinal cord CSA 24 has been described as uneven across the cord length, with most pronounced changes detected in the upper cervical portion of the MS spinal cord. However, these findings were based on relatively selective sampling strategies 12, 13, 22, 23, 24, sometimes leading to surprising results, such as the complete lack of GM atrophy after a mean disease duration of 17 years 24 a finding challenged by MRI evidence suggesting MS spinal cord GM atrophy as a strong predictor of disability 48. Finally, whilst the role of inflammatory demyelination for axonal damage and loss in acute MS is well established 16, 52, the association of axonal loss with demyelination in chronic MS has remained unclear, with some groups detecting no difference in axonal counts between lesions and nonlesional spinal cord 37.

To provide more definitive pathology indices and to explore the potential of spinal cord CSA as a predictor of axonal loss, we comprehensively sampled whole *post mortem* spinal cords from pwMS and controls focussing our analysis on: (i) axonal loss in the CSTs (large and small diameter fibers), (ii) presence and degree of GM and WM demyelination and atrophy, and (iii) their association with CSA.

## MATERIALS AND METHODS

Whole *post mortem* spinal cords of pwMS and control subjects who died of nonneurological conditions were used. Tissues had been donated through the donor scheme of the UK Multiple Sclerosis Tissue Bank (Research Ethics Committee reference number 08/MRE09/31). A single case (MS0) was provided by Professor Joanne Martin through the tissue donor programme of The Royal London Hospital (Barts Health NHS Trust, London, UK). All tissues were fixed in 4% formaldehyde solution. Clinical notes were reviewed to obtain demographics, disease onset and course and to estimate the degree of disability. The quality and the preservation of each spinal cord was macroscopically assessed at the beginning of the study by a neuropathologist (FS) to exclude any obvious compression or artefactual damage due to tissue handling. We aimed at including a similar number of samples donated by women and men.

### Tissue sampling and immunohistochemistry

**Figure 1:**
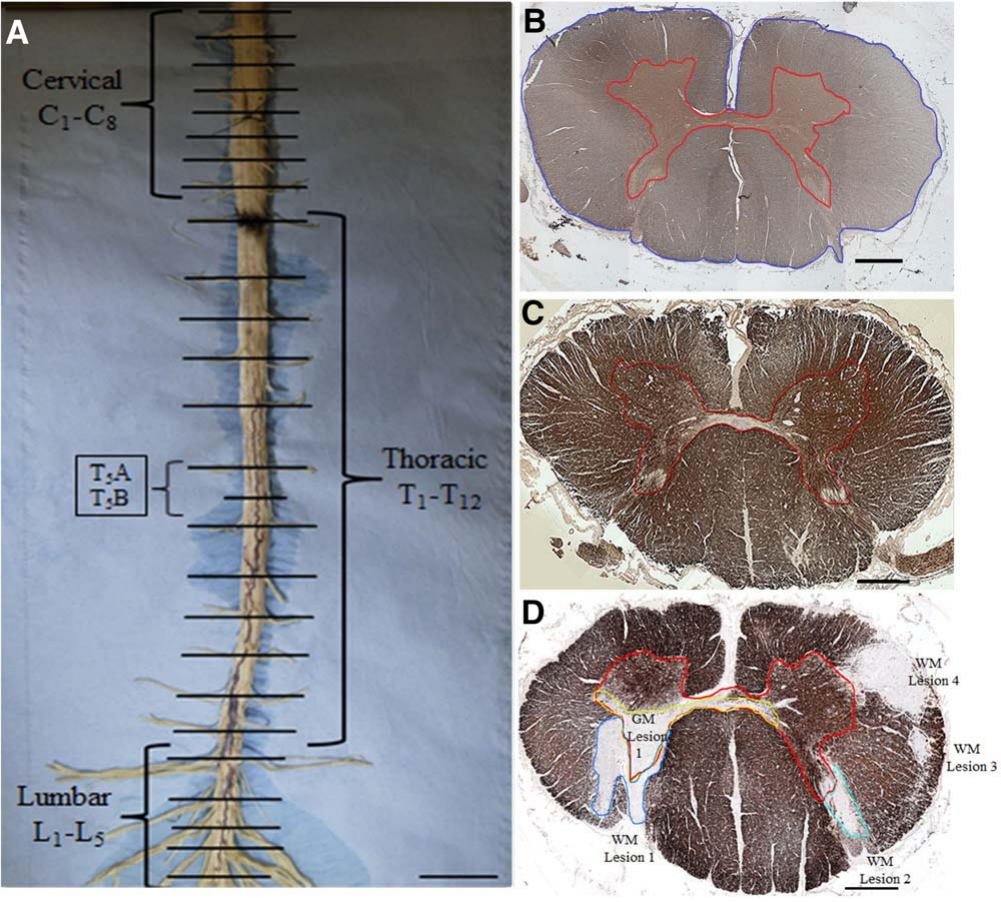
Tissue sampling and quantification of cross sectional area and demyelination. (**A**) Spinal cord dissection: after identifying and numbering nerve roots, pictures were taken and the distance between thoracic level 2 and lumbar level 5 recorded. Axial blocks were then dissected at each nerve root level. At the thoracic level an additional block was dissected between nerve roots (see examples T5A and T5B). Scale bar: 1.5 cm. (**B**) Control cervical cord section immuno‐stained for SMI‐31 at ×4 magnification. The gray matter (GM) is outlined in red and the cross‐sectional area (CSA) in blue. White matter (WM) was calculated as the difference between CSA and GM area. (**C**) Control upper thoracic cord section at ×4 magnification stained for myelin basic protein (MBP). GM borders are outlined in red. (**D**) MS upper thoracic cord section stained for MBP. Four WM (WM Lesions 1–4) and one GM lesions (GM Lesion 1, green contour) were manually outlined. Scale bar: 1 mm.

Spinal cord levels were identified based on the presence of the thinnest nerve root at thoracic level 1. The remaining nerve roots were subsequently identified and marked. Each cord was then dissected axially to result in at least one tissue block per available nerve root level (Figure 1A). Each tissue block was then marked with tissue dye to retain information on its spatial orientation and processed for embedding in paraffin wax. Of each tissue block 10‐μm thick sections were cut using a Shandon Finesse ME+ microtome (ThermoScientific, UK) Care was taken to cut them in a plane perpendicular to the anterior spinal artery. The sections were mounted on Superfrost+ slides (VWR, UK) and left in a 60°C oven overnight. The distance between the exit of the second thoracic (T2) and fifth lumbar (L5) pair or nerve roots was measured to be used as a “proxy” of total cord length, for the statistical analysis of confounding by cord length (see below).

Serial sections of each block were stained for H&E, phosphorylated neurofilaments (SMI‐31, mouse monoclonal, 1:1000, Abcam, UK) and myelin basic protein (MBP, SMI‐94, mouse monoclonal, 1:100, Covance, Princeton, NJ, USA) following a modified protocol 21, 51. To assess microglial activation and macrophages a select number of sections was also stained for CD68 (mouse monoclonal, 1:100, Abcam, UK). After cutting, sections were dewaxed in xylene and rehydrated in industrial methylated spirit (IMS) and then in tap water. After antigen retrieval was performed for 10 minutes in citrate buffer at pH6, the sections were left to cool before undergoing blocking with 2.5% normal horse serum (Vector Labs, Burlingame, CA) and avidin/biotin block (Thermo Fisher Scientific, Waltham, USA). Primary antibodies were incubated for 60 min at room temperature followed by appropriate biotinylated secondary antibody incubation for 30 min, which were detected using the ABC method (Vector Labs, Peterborough, UK). 3, 3′‐diaminobenzidine (DAB) was applied for 5 min followed by counterstaining with hematoxylin. Sections were mounted in Di‐N‐Butyle Phthalate in Xylene (DPX, VWR, UK) and left to dry. To enable direct comparison of our results with previous work by others 12 we repeated the analysis of axonal density between control and MS using only two randomly selected blocks per anatomical region.

### Area measurements

**Figure 2:**
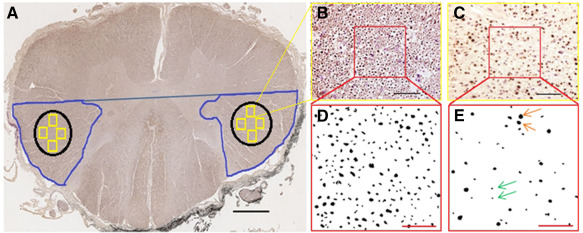
Assessment of the lateral cortico‐spinal tract area and axon counting. (**A**) The area of the lateral cortico‐spinal tract was manually outlined on SMI‐31 immuno‐stained sections (blue) at ×4 magnification as previously described by DeLuca and coworkers. An area of interest (AOI, black) was then placed well inside the anatomical boundaries of the cortico‐spinal tract. Within the AOI four frames (yellow squares) were then randomly cast and images acquired at ×40 magnification. Scale bar: 1 mm. These SMI‐31 images of control (**B**) and MS (**C**) spinal cord were opened using ImageJ. One counting field (120 μm × 120 μm, red squares) was placed inside each image. Scale bar: 50 μm. Magnified counting frames were converted into black & white (8 bit) of control (**D**) and MS (**E**) cortico‐spinal tract, and axons counted at ×40 magnification. Scale bar: 25 μm. Orange and green arrows indicate large and small diameter axons, respectively.

Images of cross‐sectional whole tissue blocks were acquired using a 4× objective (Figure 1B–D) using a standard microscope (Nikon eclipse 80i) equipped with a motorized stage (mbf bioscience, Williston, USA) and saved as TIFF files. Images of sections stained for SMI‐31 were opened with ImageJ (version 1.47; http://imagej.nih.gov/ij/) to manually outline and measure the following: (i) the CSA, (ii) the areas of GM and WM and (iii) the area of the lateral cortico‐spinal tract (aCST; Figure 2A). The latter was outlined following anatomic criteria: a horizontal line extended laterally from the most posterior part of the gray matter commissure out to the lateral border of the cord, which was used to define the anterior border. The outer margin of the spinal cord and the dorsal horn were used as the lateral and medial borders, respectively 12, 50, 51.

### Measurement of demyelination

Where present, GM and WM demyelination were manually outlined on MBP images at 4× magnification and their area measured. The total area of GM and WM demyelination in each block containing lesions was calculated as the sum of all lesion areas of the respective tissue type in the section (Figure 1D). GM and WM demyelination were then expressed as percentages. Only tissue blocks affected by WM demyelination were used for analysis of the relationship between demyelination and axonal loss.

### Assessment of axonal count, density and area

Using SMI‐31 immuno‐stained sections, four images at ×40 magnification were randomly acquired from both the left and the right cortico‐spinal tracts of each tissue block and saved as TIFF files. Care was taken that these images, which were subsequently used for quantifying the number of axons, were placed well within the acknowledged boundaries of the CST (Figure 2A). Each image was then opened and a counting frame (sized: 120 μm × 120 μm) applied (Figure 2B,C). After adjusting the white balance and transforming the area outlined by the counting frame to 8‐bit black and white, the images were saved and axons were detected using ImageJ applying an object detection threshold to quantify the number of large (sized >3 μm) and small (sized ≤3 μm) diameter axons separately 12, 37 (Figure 2D,E). To verify the validity of this approach, *n* = 31 images were randomly selected and the number of axons manually detected. The coefficient of variation between manual counting and thresholding technique was then calculated as the square root of the mean variance divided by the mean of the measurements. To measure the axonal area in each CST, the saved 8‐bit, black and white image used to count the number of axons was opened and the area of each axon measured using the “analyze particles” application of ImageJ. Threshold was set to no threshold (0‐Infinity).

Axonal density in each CST was calculated as the sum of large and small diameter axons divided by the total area of the four counting fields expressed as the number of axons/mm^2^. Given no significant difference was detected between left and right CST axonal densities, the density of axons per tissue block was then expressed as a mean of left and right for all the subsequent statistical analysis. The proportional counting of axons, as axonal density, rather than total axonal count, was used as a measure of axonal pathology since the total count may simply reflect the total size of structures rather than axonal loss. To measure the total axonal area in each CST the axonal area in all four images was averaged.

Reproducibility of our histological measurements was verified by repeating three times the quantification of axonal density in one tissue block at each cord level (cervical, thoracic and lumbar) in six cases (three MS and three controls) by N.P. The coefficient of variation (CoV) was calculated as the square root of the mean variance, divided by the mean of the measurements.

### Analysis of focal demyelination and axonal loss

**Figure 3:**
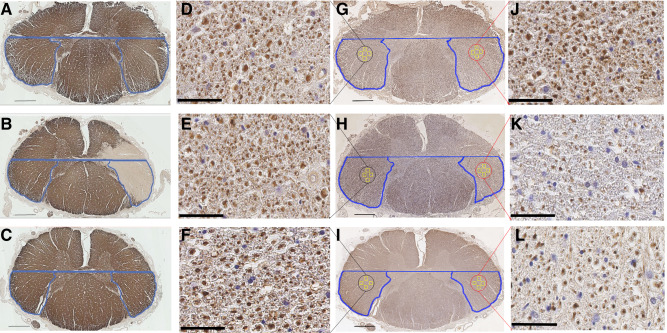
Focal demyelination and axonal loss in the spinal cord cortico‐spinal tract. Myelin basic protein (MBP) stained sections showing the nonlesional CSTs on the blocks directly above (**A**) and below (**C**) the isolated lesion, as well as the demyelinated CST on the level of the plaque (**B**). Sequential sections stained with SMI‐31 are shown for the three levels in g to i respectively in low magnification. Red circles show the CST boundaries on the homotopic, ipsilateral side of the lesion, while black circles show the CST boundaries on the contralateral, functionally identical, side of the lesion. The four yellow squares in each CST represent the four counting fields cast in each CST as described in the methods and Figure 2, where axonal density was counted. Scale bar: 1 mm. Examples of SMI‐stained images from one of the four counting fields on the contralateral side of the lesion are shown on high magnification in **D**, **E** and **F** for each of the three regions. **K** shows one example counting field from the lesional CST, while **J** and **L** correspond to the counting field examples directly above and below the lesional CST respectively, on the ipsilateral side of the lesion. Scale bar: 50 μm.

Blocks containing “isolated” WM lesions were obtained irrespective of the spinal cord proportion (cervical, thoracic, lumbar), and the following criteria applied: lesions were considered isolated if the CSTs appeared normally myelinated in at least five adjacent blocks (each approximately ∼0.5 cm thick) above and below the lesion. Regions of interest were then placed in the lesion, and (i) corresponding ipsilateral areas of nonlesional tissue that included the pathway of the CST in the block immediately above and below the lesion, as well as (ii) homotopic contralateral areas of nonlesional tissue at each corresponding level (lesion block, above lesion block, below lesion block). Axonal density and area were assessed as described above, and compared amongst groups (Figure 3). Fifteen control sections were randomly selected from controls and their axonal density and area compared with the three aforementioned isolated lesion groups.

### Statistical analysis

Differences between patients and controls were examined using linear mixed models with the measure being compared as the response variable and a fixed effect group indicator; fixed effect cord region indicators were included in all models, and other potential confounders (age at death dichotomized at <66, 66+ years to give roughly equal numbers, gender and cord length, dichotomized at <29, 29+ cm) were included singly as fixed effect covariates. These models used the cord slice as the unit of analysis, with a random subject intercept to account for the ownership of slices by subjects. An advantage of the mixed model is that it enables all available cord slices to be used in the analyses. Possible variations in patient vs control differences by region were examined by adding a group × region interaction term to the models. Linear mixed models were also used to investigate associations between cord measures and potential confounders. Residuals were examined to check model assumptions, as a result of which axonal density was log transformed to improve residual normality and homoscedasticity; this has two consequences: i) the calculated estimates and their confidence intervals represent proportional, not absolute loss; ii) it suggests that proportional loss in axonal density may be a more reliable description of patient vs control difference than absolute loss. Restricted maximum likelihood estimation (REML) was used except where there was evidence of residual heteroscedasticity, when maximum likelihood was used with robust standard errors. Analyses were performed in Stata 13 (Stata Corporation, College Station, Texas, USA), all measures were reported as average ± standard deviation (SD), except where noted. Significance is reported at 5%, unless otherwise indicated.

## RESULTS

### Subjects

*Post mortem* tissue of eighteen subjects, thirteen (seven women, six men) with MS and five people (three men and two women) with no evidence of neurological disease, who donated their CNS to the UK MS Tissue Bank, was used (Table 1). The mean age at death of pwMS was 65 ± 11 years and for healthy controls 82 ± 7 years. Disease duration in pwMS was 29 ± 11 years. None of the patients had been treated with disease modifying drugs. Although the clinical information on disability status was not available for four cases, all other patients were wheelchair bound and therefore had an EDSS of ≥7. All patients had secondary progressive MS.

**Table 1:** Clinical details of all cases.

Case	Sex	Age at onset (years)	Age at death (years)	Disease duration (years)	Number of slides analyzed
C1	Female	–	79	–	24
C31	Male	–	76	–	17
C55	Female	–	92	–	25
C61	Male	–	81	–	17
C75	Male	–	82	–	35
MS0	Female	NA	52	NA	28
MS443	Male	NA	79	NA	32
MS447	Female	37	51	14	11
MS454	Male	23	67	44	16
MS456	Male	38	59	21	28
MS465	Female	NA	66	NA	21
MS468	Female	24	55	31	17
MS471	Female	60	87	27	15
MS472	Male	30	62	33	14
MS480	Male	44	61	17	25
MS484	Female	33	81	48	18
MS550	Female	42	64	22	32
MS551	Male	30	65	35	22

NA: not available.

### Spinal cord preservation and microscopic tissue features

The wet tissue was of good quality with no macroscopically evident signs of compression and/or other artefactual damage. The samples had been fixed in formalin for 30 ± 24 months before processing. The time between death and tissue fixation was 35.5 ± 16.5 hours. The mean cord length of all spines was 27.2 ± 4.8 cm, with controls being 28.5 ± 4.4 cm and MS being 26.8 ± 5.0 cm. The number of tissue blocks analyzed ranged from 17 to 35 in controls and from 11 to 32 in MS. In total 396 tissue blocks were used for in this study. A shrinkage factor was not established in the current experiment, however a recent study pursued in our lab investigating cortical MS pathology revealed an average tissue shrinkage factor of 30% 8, 38, which is virtually identical to a previously reported factor in the spinal cord 12.

### Assessment of cellularity

**Figure 4:**
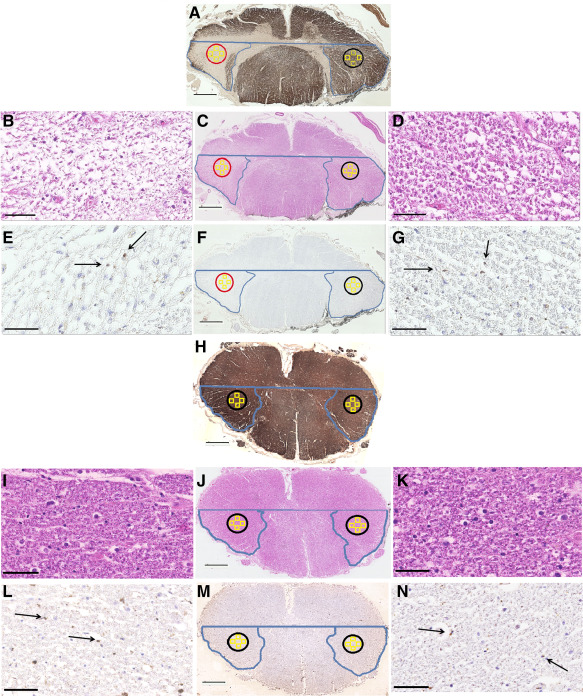
Assessment of cellularity and inflammation in control, nonlesional and lesional CSTs. Low magnification images of MBP (**A** and **H**), H&E (**C** and **J**) and CD68 (**F** and **M**) show the staining patterns in MS and control tissue respectively, while a lesional CST is shown on the left highlighted in the red circle and a nonlesional CST is shown in black on the right side of the MS image on panel **A**. Scale bar: 1 mm. High magnification images of H&E are shown **B** and **D** for the lesional and nonlesional CSTs in MS respectively, while control images are shown in **I** and **K** on the left and the right normal‐appearing CST tissue. Examples of CD68 staining in high magnification for the plaque and normal‐appearing CST are shown in **E** and **G** respectively, while control staining is shown in **L** and **N**. Scale bar: 25 μm.

H&E and CD68 (immuno‐) stained sections were used for inspection of MS and control tissue. Characteristics of the cellular composition were assessed in areas that included the CST (Figure 4). Whilst within MS lesions cellularity was decreased compared to the surrounding nonlesional tissue, the latter was indistinguishable from control tissue with no change in cellularity suggestive of inflammation. CD68 immuno‐staining did not reveal any difference between lesional and nonlesional MS tissue. Against this backdrop, no systematic assessment of inflammatory cells was undertaken.

### Reproducibility of histological indices

The CoV between manual counting and thresholding technique to quantify axonal counts was 16% for large, and 8% for small diameter axons. The CoV of the aCST measurement, plotted as an average per slide between the left and right CSTs, and combining all spinal levels for both patient and control tissue, was 10%. When separating the same measurement by level and testing for significant differences the CoV was 10% at cervical, 11% at thoracic and 4% at lumbar level with no evidence of patient vs. control difference in measurement error (*P* = 0.35). The CoV for the axonal density, when plotted as an average combining all regions and both patient and control cases, was also 10%. A separate analysis by level showed a significantly lower variability at the cervical level (6%, *P* = 0.0008) when compared to thoracic (13%) and lumbar (10%). There was no evidence of patient vs. control difference in measurement error (*P* = 0.829).

### Spatial distribution of tissue loss along the multiple sclerosis spinal cord

**Figure 5:**
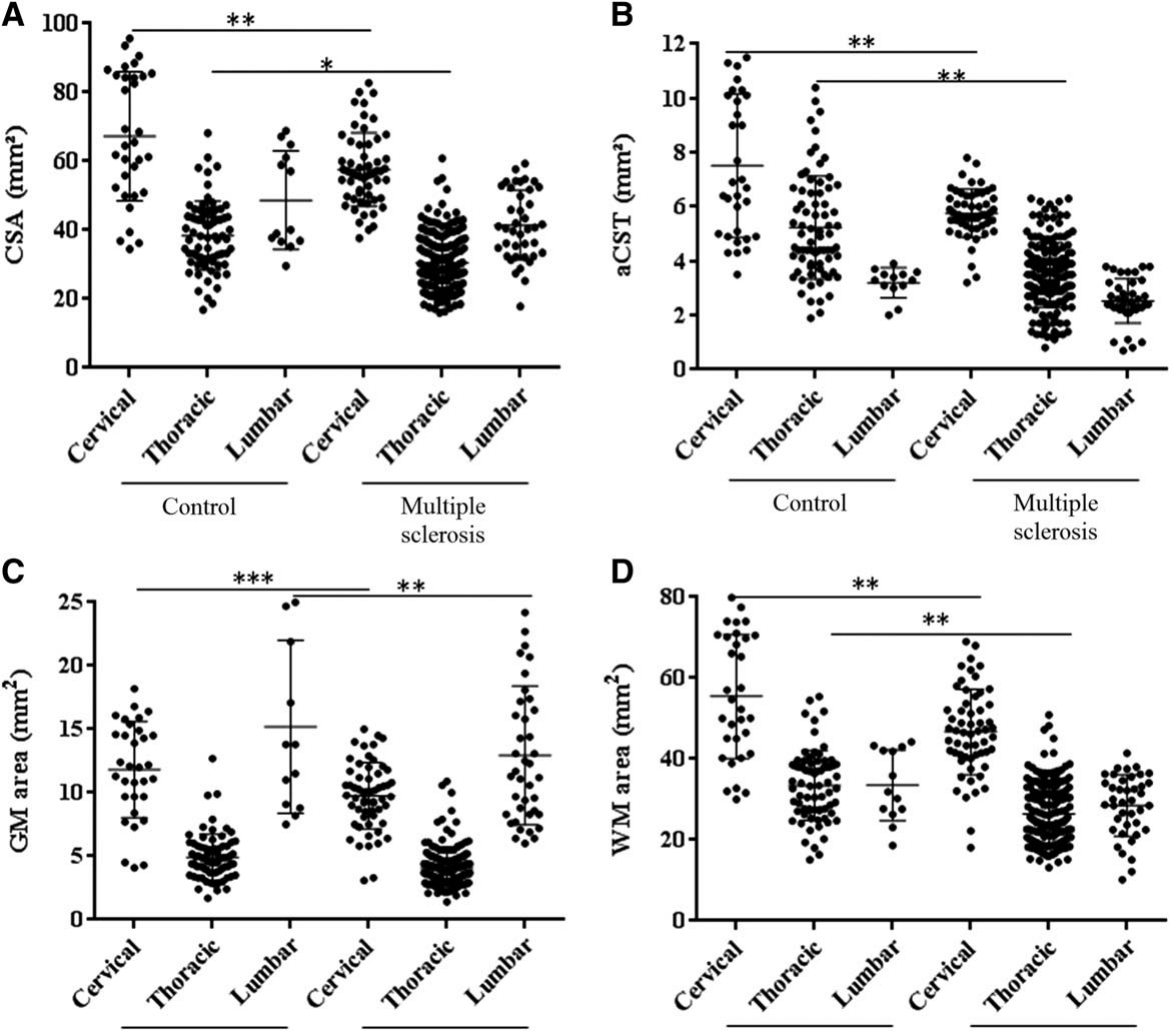
Area indices across spinal cord levels in multiple sclerosis and controls. (**A**) Cross sectional area (CSA) in MS was reduced at the cervical, thoracic and lumbar (*P* = 0.055) levels. (**B**) The area of the lateral cortico‐spinal tract (aCST) was reduced at the cervical and thoracic levels, with trend difference at the lumbar level (*P* = 0.099). (**C**) The gray matter (GM) area was reduced in MS at the cervical and lumbar levels. (**D**) The white matter (WM) area was reduced at the cervical and thoracic levels. Each dot represents one tissue block. Error bars represent standard deviations and stars indicate significance levels (***P < 0.001; **P < 0.01; *P < 0.05). See also Table 2.

Table 2 shows unadjusted patient vs control differences by spinal cord levels. The MS spinal cord CSA was lower than control by between 19% and 24%, quite small proportional variation between cord levels; however, there was borderline significant evidence, *P* = 0.055, of variation in absolute differences by region, with the largest absolute loss in the cervical region (Figure 5A).

**Table 2:** Area measurements [mm^2^], relative and percent difference across spinal cord levels between multiple sclerosis and controls.

	Patients (mean ± SD) [mm^2^]	Controls (mean ± SD) [mm^2^]	Absolute difference [mm^2^]	% loss	95% CI for absolute difference	*P* value for absolute difference
**CSA**						
Cervical	55.7 ± 10.7	69.9 ± 18.7	−14.2	20.3	−23.7, −4.7	0.003
Thoracic	31.0 ± 8.5	40.5 ± 9.9	−9.5	23.5	−18.5, −3.1	0.043
Lumbar	41.2 ± 10.1	51.0 ± 14.3	−9.8	19.2	−19.9, 2.1	0.055
**aCST**						
Cervical	5.5 ± 0.9	7.3 ± 2.6	−1.8	24.8	−3.0, −0.6	0.003
Thoracic	3.5 ± 1.2	5.5 ± 1.9	−2.0	36.5	−3.2, −0.9	0.001
Lumbar	2.6 ± 0.8	3.7 ± 0.6	−1.1	29.5	−2.4, −2.0	0.099
**GM area**						
Cervical	9.9 ± 2.6	12.5 ± 3.7	−2.6	20.8	−4.3, −0.8	0.005
Thoracic	4.1 ± 1.5	5.2 ± 1.8	−1.1	21.2	−2.6, −4.3	0.160
Lumbar	12.9 ± 5.4	15.4 ± 6.5	−2.5	17.0	−4.6, −0.4	0.019
**WM area**						
Cervical	46.5 ± 10.5	57.4 ± 15.1	−10.9	19.0	−18.9, −2.9	0.007
Thoracic	26.8 ± 7.4	35.2 ± 8.6	−8.4	23.9	−16.2, −0.6	0.034
Lumbar	28.3 ± 7.5	35.6 ± 8.4	−7.3	20.5	−15.7, 1.0	0.086

CSA = cross‐sectional area; aCST = cortico‐spinal tract; GM = gray matter; WM = white matter.

aCST was decreased in the MS spinal cord by between 25% and 37% (Table 2). There was significant evidence of variation in absolute differences across spinal cord levels, *P* = 0.017. The greatest absolute reduction was observed at thoracic, followed by cervical level (Figure 5B).

GM area differences across the spinal cord showed a reduction in MS spinal cord ranging from 17% to 21%. There was only borderline significant evidence that the absolute differences varied across the three levels of the neuraxis, *P* = 0.0515. All values are shown in Table 2 and plotted in Figure 5C.

WM area across the MS spinal cord was reduced by 19% to 24%. There was no evidence of variation in absolute differences by region (*P* = 0.165). All values are shown in Table 2 and Figure 5D.

Adjustment for age, gender or cord length did not materially alter estimated differences.

### Axonal loss affects all spinal cord levels equally and is independent of axonal diameter

**Figure 6:**
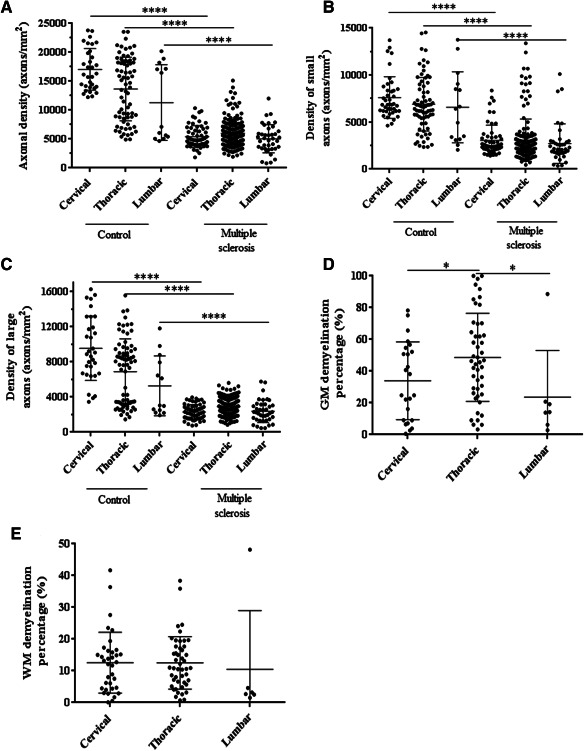
Axonal loss and demyelination in the multiple sclerosis spinal cord. Axonal density was reduced in MS spinal cord when including all (**A**), small diameter (≤ 3 µm) (**B**) and large diameter (>3 µm) (**C**) axons across all levels. (**D**) The degree of GM demyelination was higher at the thoracic level when compared to the cervical and lumbar levels. (**E**) No significant difference was detected in the extent of WM demyelination between spinal cord regions. Each dot represents one tissue block. Error bars represent standard deviations and, stars indicate significance (****P < 0.0001; *P < 0.05).

Axonal density in the MS cortico‐spinal tract was significantly reduced by between 57% and 62% (Tables 3 and 4, Figure 6A), with no evidence of variation in this loss across spinal cord levels (*P* = 0.145). These estimates were obtained using the log transformed density, which better satisfied normality assumptions than the raw density. There were similar reductions in both large and small diameter axons in MS spinal cords of 61% (large) and 60% (small), with no evidence that the proportional reduction in small diameter axons is greater than in those with a large diameter, *P* = 0.973 (Figure 6B,C). These estimates were not materially affected by adjustment for age, gender or cord length.

**Table 3:** Differences in axonal density between normal control and multiple sclerosis.

	Controls (mean ± SD)	Multiple sclerosis (mean ± SD)	Difference	95% CI	*P* value
**Axonal density**	[axons/mm^2^]	[axons/mm^2^]	[%]		
Cervical	17051 ± 3607	5403 ± 1866	−62	43%,74%	<0.001
Thoracic	13866 ± 5715	6049 ± 2496	−57	37%, 71%	<0.001
Lumbar	11307 ± 6542	5043 ± 2458	−62	43%, 75%	<0.001

**Table 4:** Differences in percent demyelination between gray and white matter in multiple sclerosis.

	Gray matter (mean ± SD)	White matter (mean ± SD)	Absolute difference	*P* value
**Demyelination**	[%]	[%]	[%]	
Cervical	33.9 ± 24.4	12.6 ± 9.6	21.3	0.052
Thoracic	47.6 ± 28.3	12.5 ± 8.3	35.1	<0.001
Lumbar	23.7 ± 29.4	10.5 ± 18.5	13.2	0.130

SD= standard deviation; CI= confidence interval.

Assessing the extent of axonal loss using only two blocks per anatomical region showed that the axonal density was reduced by 64% at the cervical, 66% at the thoracic and 64% at the lumbar level. Only marginal, not statistically significant, difference in the percent change of axonal density between control and MS was detected compared to the analysis including all tissue blocks (see above).

### Demyelination is more extensive in gray than white matter

When analyzing only tissue blocks that contained “any” area of GM demyelination its percentage ranged from 24% to 48% (Figure 6D, Table 4), with evidence of a significant variation across the three spinal cord levels (*P* = 0.001), with the most extensive GM demyelination affecting the thoracic level. Thoracic GM demyelination was significantly greater than at both cervical (*P* = 0.001) and lumbar (*P* = 0.018) levels.

Focusing on tissue blocks that contained “any” area of WM demyelination only its percentage ranged from 11% to 13%, with no evidence of variation across the three cord levels (*P* = 0.936). (Figure 6E, Table 4).

Across the MS spinal cord GM demyelination as a percentage of total GM was more extensive than WM demyelination; including all cord levels the mean percent difference between GM and WM demyelination was 26% (95% CI 15, 36, *P* < 0.001). Region‐specific percent differences between GM and WM demyelination are shown in Table 4.

### Axonal density correlates with disease duration

**Figure 7:**
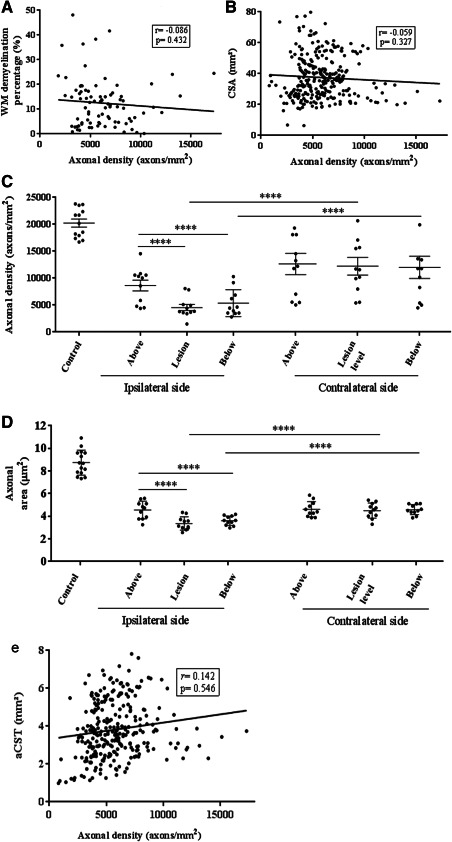
Association between axonal density and area and focal cortico‐spinal tract (CST) demyelination, axonal density and total white matter demyelination and cross‐sectional area. Overall, white matter (WM) demyelination did not correlate significantly with axonal loss (**A**). However, focal demyelination affected axonal density at the lesion site (*P* < 0.0001) and in nonlesional CST right below the lesion (*P* < 0.0001). No change was detected in nonlesional CST right above the lesion, or the homotopic normal appearing white matter on the contralateral side at any level (**C**). Axonal density in controls was significantly higher compared to nonlesional CST above the lesion (*P* = 0.035) as well as lesional CST, and the nonlesional CST directly below (*P* < 0.001, Table 6). Axonal area was also affected by focal demyelination locally (*P* < 0.0001) and on the nonlesional section right below the lesion (*P* < 0.0001), while no difference was observed between nonlesional CST above the lesion, or the homotopic normal appearing white matter on the contralateral side (*P* = 0.224). Axonal area was significantly higher in the control CSTs compared to all three groups (lesion, and both locations directly above and below it (*P* < 0.001, Table 6). No correlation was detected in the MS spinal cord between axonal density and (**B**) CSA or (**E**) “area of CST” (see methods for explanation). Error bars represent standard deviations and, stars indicate significance.

In MS, after adjusting for region, higher axonal density was borderline significantly associated with shorter disease duration (*P* = 0.055). Disease duration did not correlate with aCST, GM or WM area. After adjusting for subject group and region, no significant association was detected between cord length as well as age at death and (i) axonal density, (ii) cortico‐spinal tract, and (iii) GM or WM areas. No significant correlation emerged either between axonal density and (i) aCST and (ii) CSA (Figure 7A,B).

### Axonal loss is strongly associated with focal white matter demyelination, however overall lesion burden does not reflect this

Including all blocks in which WM demyelination was detected, i.e., irrespective of lesion location, and without applying the strict rules for “isolated” lesions, no association emerged between the total extent of WM demyelination and axonal loss (Figure 7A). In 10/13 cases included in this study, 11 “isolated” WM lesions were identified. Focusing on the analysis of CST fibers affected by an isolated lesion revealed that axonal density within and below these lesions was lower by 51% and 42%, respectively, compared to the density measured above the lesions (*P* < 0.001). Homotopic contralateral areas showed no difference between the two sides above the lesion level, however significant decrease at the lesion level (–48%) and below (–42%; *P* < 0.001). A random selection of sections from all available control cases revealed that a 30% decrease in axonal density in the CSTs directly above the isolated lesions (*P* = 0.035), was accompanied by an additional axonal loss of 65% and 56% in the lesion and the CST directly below it compared to control sections respectively (*P* < 0.001, Tables 5 and 6, Figure 7C).

**Table 5:** Mean axonal density and area in the lesion site and the two sites corresponding to the normal‐appearing white matter directly above and below the lesion site on the ipsilateral side of the lesion and the corresponding contralateral sites on the same blocks as well as on a random selection of fifteen control sections.

	Ipsilateral mean(SD)	Contralateral mean(SD)	Absolute difference	% change	95% CI for absolute difference	*P* value for absolute difference
**Axonal density**	[axons/mm^2^]	[axons/mm^2^]	[axons/mm^2^]	[%]		
Above lesion	10663(3919)	10468(4902)	567	5	−1514, 2649	0.593
Lesion	5174(2325)	10604(4360)	−5058	−48	−7139, −2976	<0.001
Below lesion	6227(3300)	11463(5171)	−4864	−42	−6945, −2782	<0.001
Control	16606(4881)	–	–	–	–	–
**Axonal area**	[μm^2^]	[μm^2^]	[μm^2^]	[%]		
Above lesion	4.60 (0.78)	4.56 (0.68)	0.04	1	−0.33, 0.41	0.816
Lesion	3.36 (0.58)	4.50 (0.67)	−1.14	−25	−1.51, −0.77	<0.001
Below lesion	3.67 (0.41)	4.46 (0.44)	−0.79	−18	−1.16, −0.42	<0.001
Control	8.74 (1.10)	–	–	–	–	–

**Table 6:** Differences in axonal density and area on the ipsilateral side of the lesion and control CSTs compared between the three positions, above the lesion, inside the lesion and below the lesion.

Ipsilateral side	Absolute difference	% Change	95% CI for absolute difference	*P* value for absolute difference
**Axonal density**	[axons/mm^2^]	[%]		
Lesion vs above lesion	−5489	−51	−7248, −3730	<0.001
Below lesion vs above lesion	−4436	−42	−6195, −2677	<0.001
Below lesion vs lesion	1053	20	−706, 2812	0.241
Above lesion vs control	−5047	−30	−9743, −351	0.035
Lesion vs control	−10768	−65	−14648, −6887	<0.001
Below lesion vs control	−9356	−56	−13598, −5114	<0.001
**Axonal area**	[μm^2^]	[%]		
Lesion vs above lesion	−1.25	−27	−1.75, −0.74	<0.001
Below lesion vs above lesion	−0.93	−20	−1.44, −0.43	<0.001
Below lesion vs lesion	0.31	9	−0.19, 0.82	0.224
Above lesion vs control	−3.87	−44	−4.79, −2.95	<0.001
Lesion vs control	−5.31	−61	−6.14, −4.47	<0.001
Below lesion vs control	−4.96	−57	−5.77, −4.15	<0.001

The differences, CIs and *P*‐values were obtained from mixed models accounting for the cord/slice hierarchical structure: thus they do not match the difference in raw means.

Differences in axonal area followed a similar pattern to axonal loss, as described above, with axonal area within and below the isolated lesions showing a reduction of 27% and 20%, respectively, compared to NAWM above the lesions (*P* < 0.001). Axonal size reduction was also observed in lesions and the NAWM directly below them when compared to the homotopic contralateral areas by 25% and 18%, respectively (*P* < 0.001). Axonal area was significantly lower in the CSTs above the lesion compared to control sections by 44%, while an additional decrease in axonal size was seen when comparing control CSTs to lesional CSTs and the NAWM directly below them by 61% and 57% respectively (*P* < 0.001, Tables 5 and 6, Figure 7D).

## DISCUSSION

Although axonal loss in MS has been described as a pathological hallmark contributing to functional disability, results reporting the extent of axonal pathology have been inconsistent. Whilst this may be due to differences among tissue collections, other limitations including sampling bias may have contributed to variable results reported 6, 12, 19, 37, 50. We therefore employed a comprehensive sampling strategy to investigate axonal loss and its relationship with volume changes and demyelination across the whole spinal cord in MS.

Our CSA measurements indicate that in chronic MS spinal cord atrophy of approximately 20% occurs and that this atrophy evenly affects all levels of the spinal cord. No material difference was detected between the spinal cord levels when analyzing GM and WM atrophy separately. The WM loss detected was between 15% and 21% whilst GM loss was approximately 17% at cervical and lumbar levels, with similar trend difference at the thoracic level. Area reduction was also detected specifically affecting aCST, though this was statistically significant only at the cervical and thoracic levels. The degree of aCST reduction at the thoracic level (37%) suggests that the loss of CST fibers may be disproportional to the overall spinal cord atrophy, which affected all areas equally.

Axonal density was reduced by 57–62% across all levels of the spinal cord. This degree of axonal loss across the entire spinal cord is remarkably similar to the loss reported by Tallantyre *et al* 50 and Bjartmar *et al* 6; approximately twice the loss reported by DeLuca *et al* 12; and about 1/3 higher compared to Ganter *et al* 19. The cohorts of MS cases used in these four studies had an average disease duration of 25, 24, 17, and 14 years, respectively, while it was 29 years in our cohort, suggesting that axonal loss accrual is indeed proportional to clinical disease duration. This interpretation is also supported by the fact that in our cohort, and after adjusting for anatomical and clinical confounders, the longer the disease duration the lower was axonal density within the CSTs. The dichotomous distribution of axonal densities in the control population can be explained by a single control being different from the remainder. Although we were unable to establish the cause of this difference, excluding this control did not lead to a significant difference in the overall result. The case was therefore included for greater statistical power.

The spinal cord has been a focus of MRI studies for some time, and several groups reported strong association between loss of CSA and disability 5, 7, 36, 39, 43. MRI‐derived spinal cord atrophy indices are therefore increasingly being used in clinical studies 32, including treatment trials (NCT00731692), of people with CDD due to MS. The degree of CSA loss reported in *in vivo* studies (for example, 28% in 36) has been of similar magnitude compared to our results (–20%), and an earlier study (–25% in 6) using *post mortem* samples. Regarding the contribution of GM and WM to CSA changes, results have been equivocal: Whilst an earlier *post mortem* study reported significant volume loss of the WM, but only a minor trend reduction in the GM 24, we detected no proportional difference between GM and WM, in line with work by Bjartmar *et al* 6. Differences in disease duration between studies may account for variable results. Whilst Bjartmar *et al* used samples of pwMS who had the disease for a very similar time as in our study 6, samples in Gilmore *et al* 24 were from pwMS who had MS for, on average, 12 years less 24. Of interest, recent *in vivo* evidence suggests GM atrophy is more strongly linked to the degree of disability 33, 49.

Despite sufficient statistical power, no association emerged in our study between CSA and axonal density (Figure 7). The difference between CSA reduction (20%) and axonal loss (about 60%) suggests spinal cord atrophy significantly underestimates the degree of axonal loss, a putative key substrate of CDD. These findings corroborate observations in animal models of MS in which the time course was monitored 26. Whilst previous studies using human *post mortem* MS samples have investigated CSA changes 24 and axonal loss 12, 19, 37, correlation between CSA and axonal density has, to our knowledge, been explored only once before 6. In line with our results no association between axonal loss and CSA was detected.

If spinal cord volume does not provide a straightforward correlate of axonal density, other factors need to be considered. The expected degree of atrophy based on axonal loss could be offset by “space filling” tissue components such as edema, inflammation and gliosis. For example, in a clinical study of the sodium channel blocker lamotrigine pseudo‐atrophy (here: of the brain) due to anti‐edematous effects confounded the detection of a (putative) neuroprotective effect 30. However, although in our study fluid was removed as a result of tissue processing and fixation, it is unlikely that edema would account for the large difference detected between CSA and axonal density. Gliosis is a rather more likely candidate given it evidently counteracts the space reducing effect of nerve fiber loss 6, 26.

The contribution to spinal cord atrophy of nerve fiber loss affecting tracts other than the CST should also be considered 58. A mean 13% loss of axonal density in the spinal cord dorsal columns has previously been reported with marginal impact on dorsal column CSA (impact on CSA not reported) 12. Further spinal tract systems may be affected, however, their anatomical boundaries are often less well defined though impaired conduction has been reported in the spinothalamic tract 28.

As suggested by the degree of GM to CSA reduction, not only tract systems are likely to contribute to CSA.

Neuronal shrinkage and a moderate degree of spinal cord neuronal loss are likely additional candidate factors contributing to both CDD and spinal cord atrophy 23, 47. Moreover, our group recently reported loss of synaptophysin affecting both the normal appearing as well as lesional spinal cord GM. Depending on the index used, this loss exceeded 90% compared to controls, and correlated moderately with GM area 45. Whilst loss of synaptophysin in MS has previously been reported in the neocortex 55, hippocampus 14, and cerebellar dentate nucleus 2, the substantial degree of synaptic damage in the spinal cord may be an important driver of CDD, over and above axonal loss 45.

Taken together, these observations suggest that spinal cord atrophy is only partially reflective of the pathological feature considered crucial for CDD, i.e., axonal loss. Translated into the clinical setting of pwMS, rather than relying on atrophy measures (alone), there appears to be an urgent need for better techniques reflecting axonal damage in the spinal cord 20.

Contrary to earlier studies no difference in the degree of axonal loss between large and small diameter axons (*P* = 0.973) was detected, questioning the selective vulnerability of small diameter axons as previously suggested 12, 37, 51. It was not possible for us to verify whether respective data in previous studies were normally distributed. However, analyzing the entire spinal cord and applying log transformation to improve residual normality and homoscedasticity of our not‐normally distributed data indicate that axons of all sizes are equally affected. It should be kept in mind, however, that axonal diameter may decrease in the process of neurodegeneration 27, possibly due to a decrease in neurofilament gene and nerve growth factor receptor expression 44. It is therefore possible that large diameter axons in chronic MS become atrophic over the course of the disease. As a result, the number of small diameter axons detected may consist of both axons that always were of small diameter as well as large diameter axons that have become atrophic. However, although axonal swelling has been previously reported as a pathological feature of axonal injury in chronic MS 6, 17, 57, our results not only suggest that axonal area in the NAWM is over 40% smaller than in control CSTs, but that axons in lesions are yet smaller. Unlike some of the above‐mentioned studies 17, 57 reporting increased axonal volume (“swelling”), however, inflammation was minimal in our samples, suggesting differences between findings may be explained by different samples used, with axonal shrinkage being a prevalent feature in material largely containing chronic inactive lesions, such as ours.

In line with previous studies, demyelination was significantly more extensive in the GM than the WM, underlining the nature of MS as a condition that affects all CNS tissue compartments 22, 25 and evidently rendering the previously held belief that MS is a merely “WM” disease outdated. Several groups reported a lack of association between demyelination and axonal loss in the MS spinal cord, a surprising result given the (multi‐) focal nature of the disease 6, 12, 19, 37, 51. Even in studies employing a highly focussed approach, differences in axonal density within and outside lesions did not always emerge 37. While the overall plaque load in our study did not correlate with the total extent of axonal loss, we detected reduced axonal density, by over 50%, in isolated CST lesions and the associated distal nonlesional CST (Wallerian degeneration). Evidence suggests acute axonal damage 16, 52 and Wallerian degeneration 15 are characteristic features of newly emerging lesions. Our data indicate that even in the very late stage of MS spinal cord demyelination has significant impact on tract specific axonal loss distal to lesions, whereas “dying back” neuropathy, as previously suggested, seems of lesser importance 9.

The apparent discrepancy between analyzing our data using (i) all blocks containing demyelination and their associated axonal density and (ii) only blocks containing lesions that affect the CST may have been due to at least two factors. First, in the former analysis we included the total area of demyelination, i.e., including areas outside the CST, whilst axonal density was established in the CST only. This may have masked the effect of focal demyelination on axonal density. Second, although we comprehensively examined the entire length of the spinal cord, we were unable to account for the potential effects of supra‐spinal (brain) tissue pathology on spinal cord axonal density.

The effect of focal demyelination on CST axonal loss in our and previous studies 50 was quite different from those reported by Lovas and coworkers who did not detect reduction in axonal density, but in axonal thickness 37, 51. Our data suggest differences in the methodology of quantification may explain these discrepancies. Whilst we are not aware that Lovas *et al* excluded any lesion pathology above or below their lesion of interest we confirmed the absence of tract specific demyelination in five tissue blocks above and below the index lesion in our “isolated lesion” experiment. We thereby significantly increased the likelihood that remote effects of demyelination did not bias our measurements. An additional, or alternative, explanation could be differences in lesion size with longer lesions hypothetically being more damaging, as has been described using MRI in people with optic neuritis 29. However, lesion size was not assessed, neither in our, nor to the best of our knowledge in previous *post mortem* studies. There could be merit adding such measures in the future to provide further insights into the effect of demyelination on axonal density.

The lack of a significant difference in the detected axonal loss when including only two tissue blocks per anatomical region compared to the analysis of all tissue blocks produced suggests that to investigate axonal loss *in isolation* two blocks per anatomical level appear to be sufficient. However, sampling the entire cord not only enables the total magnitude of other pathological features to be assessed, but also to rule out remote effects on specific areas of interest, such as in our “isolated” lesion analysis. Such lesions were also quite rare underpinning the merit of dissecting the entire cord, though MRI guidance may be an alternative solution as has been shown before for sampling lesions in the *post mortem* brain 10.

The association detected between axonal density and disease duration suggests time is a key determinant of clinically significant tissue damage in chronic MS 53. Although our data have been obtained using *post mortem* material, *in vivo* evidence of brain atrophy assessed using MRI suggests that a chronic “slow burning” axonopathy may be a feature of MS from the earliest stages of its evolution 11, over and above lesion‐associated acute axonal damage 16, 18, 52. This may be triggered by the inflammatory responses that drive relapses, as seen experimentally 26. However, these smouldering lesions increase with disease duration and chronicity in MS and probably contribute to worsening disability 18. Unfortunately, unlike biopsy studies 46, the nature of *post mortem* investigations often precludes exploring firm relationships between pathological findings and degree of disability.

A previous study by Androdias *et al* suggested association between axonal loss in the NAWM and the density of MHC II and CD3 positive cells in the meninges 4. However, although the degree of axonal loss in our study was comparable to that study, cellularity in our cases appeared well below the reported levels suggesting our cohort largely included chronic silent lesions without any signs of active inflammation.

In conclusion, our study provides robust evidence that after a life with MS for, on average, nearly 30 years about 60% of spinal cord CST axons are lost. It is likely this loss is an important driver of disease deterioration and permanent disability in pwMS and affects axons of any caliber throughout the length of the spinal cord. The magnitude of spinal cord atrophy, about 20%, does not reflect well the degree of axonal loss, which in itself appears strongly associated with focal demyelination. MRI indices of spinal cord volume obtained *in vivo* are likely to underestimate the extent of axonal damage thereby potentially limiting the role of such indices as a treatment outcome in clinical trials. New techniques that are more specific for the underlying tissue damage are warranted 20.

## FUNDING

This study has been supported by a grant from Barts Charity (grant # 468/1506).
